# Supplementary material for: Are carbon emissions trading and green financial instruments synergistic? -Comprehensive quantitative research based on content analysis
Source: PLoS One. 2024 Mar 7;19(3):e0298601. doi: 10.1371/journal.pone.0298601 (PMC10919601; doi:10.1371/journal.pone.0298601)
Supplement: S1 Data — (ZIP) [file pone.0298601.s001.zip › Data/地级市Z_F工作报告.zip!/╣π╬≈/║╪╓▌╩╨/└┤╫╘╣1⁄2╓┌║┼í╛┬φ┐╦╩2╛▌═°í┐_2016.docx]

各位代表：

现在，我代表市人民政府，向大会作政府工作报告，请予审议，并请各位政协委员和列席会议的同志提出意见。

一、2015年和“十二五”工作回顾

去年，是2008年以来宏观经济形势最为严峻的一年。面对极其复杂的困难挑战，我们在自治区党委、政府和市委的坚强领导下，在市人大、市政协的监督支持下，坚决贯彻落实中央和自治区的决策部署，统筹推进稳增长、促改革、调结构、惠民生、防风险等各项工作，努力化解发展中的困难和矛盾，经济运行总体平稳、稳中有进、稳中向好。

一年来，我们千方百计稳增长。坚持把稳增长摆在首位，深入贯彻落实自治区稳增长“48条”措施，及时出台实施了《关于千方百计做好稳增长工作的实施意见》，在“优服务、扩投资、推三产、减负担、促工业、扶实体、稳农业、强支撑”等方面取得了显著成效。据统计，全市地区生产总值增长7.6%，固定资产投资增长18%，规模以上工业总产值增长10%，财政收入增长16.1%，社会消费品零售总额增长10%。

一年来，我们坚定不移调结构。着力打造以碳酸钙和新型建筑材料“两个千亿元产业”为龙头的特色工业，加快发展生态经济，产业结构转型升级扎实有效。质量效益更高，财政收入占GDP的比重上升为9.76%，一般公共预算收入增长18.7%，其中非税收入占比低于上年1.7个百分点。投资结构更优，基本建设增长18.9%，拉动全市固定资产投资增长9个百分点。民间投资占固定资产投资比重71.1%，提高0.8个百分点。工业投资增长8.8%，其中制造业投资增长14.5%；服务业投资加快，增长32%，提高14.6个百分点。产业新动能更强，“两个千亿元产业”规模以上企业产值增长33.8%，对全市规上工业总产值增长的贡献率为43.8%；服务业增长10%，创下了“十二五”以来第三产业最高增速，其中四大业态发展势头迅猛，接待游客总人数和旅游总收入分别增长18.2%和25%；贺州生态产业园开园建设，成为全区四个生态产业园之一，创建自治区高新技术产业开发区通过评审。

一年来，我们统筹城乡谋发展。城市建设更美，深入实施市容市貌综合提质三年行动，年度完成投资11.3亿元左右，生态新城核心示范工程、“一江两岸”建设开发工程等八大工程顺利推进，城市道路交通、管网、污水垃圾处理等基础设施建设进一步加强；环境风险安全隐患大清查大整治行动扎实开展，207国道平桂段扬尘污染得到有效治理，圆满完成自治区下达的PM10控制目标任务，城区大气质量持续改善，贺州水源地的保护利用得到强化。农业发展更稳，不断增强农业综合生产能力，粮食总产量达72.34万吨；加快现代特色农业发展，贺州市创建国家农业科技园区通过评审；有序推进农村土地承包经营权确权登记颁证工作，已颁证面积达11.03万亩；完成精准扶贫识别阶段任务，加快贫困村基础设施建设步伐，自治区下达的年度减贫任务全面完成。

一年来，我们加大改革促开放。体制机制更顺，“多规合一”改革试点取得良好成效，创造性地提出“1+3+X”市县规划体系编制模式，建立“多规合一”业务协作平台和项目联合审批信息管理平台，试点成果被国家发改委和环保部列为示范；取消、下放和调整审批事项213项，“先照后证”“三证合一、一照一码”等商事制度全面推行，新登记各类市场主体1.28万户，增长36.1%；新登记企业2042户，增长36.6%。“走出去、引进来”的步伐更快，《粤桂产业合作示范区发展规划》通过广西、广东两省区评审并实施，梧州海关驻贺州办事处揭牌成立，“中国贺州石材·碳酸钙工业展览会”和“第八届贺州农产品（深圳）交易会”“华润雪花啤酒（贺州）首届啤酒节”等重大活动成功举办，全年招商引资（含续建）项目到位资金288.3亿元，完成年度目标任务的102.9%。

一年来，我们夯实基础惠民生。公共财政用于民生领域支出的比重达80.7%。城镇居民人均可支配收入、农民人均纯收入分别增长6.8%和9.8%，城镇新增就业1.5万人，城镇登记失业率为2.48%，城乡差距进一步缩小。同时，为民办实事成效显著，年度六个方面重点民生项目全面完成。农村道路建设方面，建成通建制村水泥（沥青）路83.2公里，新增硬化村屯道路633公里。农村安全饮水工程方面，新建农村人饮项目171处，保障了14.9万人的饮水安全，并建成水质检测中心4座。农户住房保险工程方面，筹集491.03万元，为43.07万户农户购买了农村住房政策性保险。城镇保障性住房项目方面，开工建设2.57万套，基本建成4000套。幼儿园建设方面，新建公办中心幼儿园17所，超额完成年度任务。绿化示范村屯项目方面，建成自治区级绿化示范村屯342个、市级精品绿化村屯25个，绿化一般村屯2157个。

市三届人大一次会议召开以来的五年，是打造贺州发展“升级版”进程中具有重要意义的五年。随着2015年主要目标任务的完成，我市“十二五”规划顺利收官，为“十三五”发展奠定了坚实基础。这五年，贺州发展取得了新的重大成就。

经济综合实力跃上新台阶。地区生产总值年均增长8.4%，“十二五”末达到468.1亿元，是2010年的1.58倍。财政收入年均增长16.1%，达到47.14亿元，是2010年的2.1倍。固定资产投资年均增长22%，达到625.93亿元。金融机构存、贷款余额分别达到530亿元和310亿元，余额存贷比保持在60%以上。人均地区生产总值2.48万元，贺州经济发展进入中等收入阶段。

产业结构调整得到新优化。与2010年相比，三产占比提高了5.2个百分点；规模以上工业总产值达到422.6亿元，年均增长19.1%，是2010年的2.37倍，其中工业园区规模以上工业总产值达302亿元，占全市规模以上工业总产值的71.5%。特别是，“两个千亿元产业”和生态健康产业异军突起，为发展提供了新动能，碳酸钙产业产值突破100亿元，重质碳酸钙粉体产品在全国市场占有率达61%，贺州成为全国最大的重质碳酸钙粉体生产基地；生态旅游业实现大幅增长，接待游客总人数和旅游总收入年均增长分别为24.96%和34.3%。粮食产量连年增长，在全国率先成为“国家级出口食品农产品质量安全示范区”全覆盖的地级市，出口蔬菜、脐橙、马蹄等农产品居全区第一。产业支撑更加坚实，贺州正加快从农业大市向新兴工业城市转变。

基础设施建设实现新跨越。城市建设大会战、综合交通基础设施大会战全面实施，广贺高速全线贯通，贵广高铁开通运营，贺州迈进了“高铁时代”，铁路运营里程达243公里，二级以上等级公路总里程达822公里，实现全部乡镇和建制村通水泥（沥青）路，群众出行更为便捷。中心城区建成区面积扩大至40平方公里，城镇化率提高了8个百分点达到43%。同时，水利、通信等保障能力大幅提升。随着基础设施建设大提速，贺州正由全区交通“末梢”向桂粤湘区域性交通枢纽迈进。

开放交流合作开启新篇章。自治区赋予我市“要发挥向东开放排头兵作用，把贺州建设成为广西对接东部和中部地区的重要门户和枢纽”的发展定位，并明确支持贺州融入珠三角经济圈。全面实施“向东开放”战略，与广东肇庆合作共建了粤桂县域经济产业合作示范区并取得积极成效，园区产值突破150亿元。引进了华润、中冶、中铝等一批央企名企合作项目，华润火电、水泥、啤酒等项目竣工投产，华润循环经济产业示范区先后被评为自治区循环经济示范园区和A类产业园区，并成为全区第一个“国家循环经济教育示范基地”。对外开放的步伐不断加快，贺州正从相对封闭的地区走向广西开放合作的前沿。

全面深化改革取得新突破。成为广西唯一的全国“多规合一”试点市，全市“一本规划、一张蓝图”的规划衔接协调机制基本建立。积极稳妥开展扩权强镇工作，实现61个乡镇“四所合一”机构改革全覆盖。17个重点镇城乡一体化示范工程有效带动新型城镇化发展。140个市本级部门公开部门预算和“三公经费”预算，行政和参公单位公车改革全面推进。县级公立医院改革工作进展顺利。完成“四大银行”升格工作，设立了东盈银行、昭平农商行和首个农村金融综合服务站等农村金融机构。组建正赢、正润、旅游实业和矿投等集团公司。各项改革全面深化，贺州发展正释放强大动力与活力。

生态文明建设发挥新优势。果断处置“贺江水污染事件”，全面开展环境隐患专项排查整治行动，并持续巩固整治成果，生态保护与建设取得了显著成效。森林覆盖率高达72.88%，是全国平均水平的3.6倍，位居全区第二。城市水域功能水质、集中式饮用水源地水质达标率均为100%，环境空气质量达到Ⅱ级标准，提前实现主要污染物排放总量控制目标要求。获得国家森林城市、中国最佳生态旅游示范城市等荣誉称号，成为全区唯一入选“全国生态保护与建设示范区”的地级市，率先在全国实现“中国长寿之乡”县域全覆盖，成为中国第一个长寿市。生态经济发展势头强劲，《贺州市生态健康产业发展规划》获得批复实施，率先在全区培育壮大了生态健康旅游、生态健康休闲、生态健康养生和生态健康晚年等四大业态。生态优势加快转化为经济和社会效益，“美丽贺州”逐渐展现在世人面前。

保障和改善民生有新作为。民生领域财政累计支出555.7亿元，占一般公共财政预算支出的78.9%。预计，城镇居民人均可支配收入、农民人均纯收入年均分别增长10.1%和13.7%，达到25595元和8181元，是2010年的1.62倍和1.9倍。累计新增城镇就业8万人，新增农村劳动力转移就业16.5万人。实施各类教育项目1672个，开展城区教育建设大会战并取得实效，新建贺州高中并实现搬迁办学，随着一批新建中小学校竣工投入使用，有效解决了城区义务教育“大班额”问题。建成村级公共服务中心、农家书屋、革命历史纪念馆、村村通广播电视乡镇无线发射台等一批公共文化基础设施。率先在全区实施“千村万户文艺惠民工程”。全民健身事业水平不断提升，贺州籍体育健儿在全国以上赛事中获得金牌34枚，涌现出了蒋惠花等国家级优秀运动员。群众医疗、养老、住房得到切实保障，建成了市人民医院综合楼等一批卫生基建项目，实现了村级卫生所全覆盖；新型农村合作医疗参合率达99.17%，城乡居民社会养老保险参保人数不断增加，累计开工建设保障性住房、棚户区和危房改造近20万套（户）。美丽贺州乡村建设成果丰硕，农村人居环境大幅改善。建成566项饮水安全工程，解决82.4万农村人口安全饮水问题。170个贫困村整村推进扶贫开发任务全面完成，预计五年累计减贫37.4万人。社会事业全面进步，贺州人民生活更加幸福！

法治政府建设展现新成效。全面推进依法行政，认真执行市人大及其常委会的决议和决定，自觉接受人大、政协和社会监督，人大代表建议和政协委员提案办结率均达100%。建立了市政府法律顾问制度和行政调解联席会议制度。坚决贯彻执行中央“八项规定”和国务院“约法三章”，深入开展党的群众路线教育实践活动和“三严三实”专题教育，廉政建设和反腐败斗争取得实效。

五年来，我们全面强化社会治理创新，做好信访、人民调解和行政调解工作，治安管控水平不断提高，“平安贺州”创建取得新成效。全市安全生产形势总体稳定，安全生产约束性指标提前完成“十二五”目标。驻梧管理、编制、保密、外侨、司法、审计、统计、税务、质监、史志、档案、民族宗教、机关事务、住房公积金、农机、供销、水产畜牧、二轻联社、水库移民、食品药品监督、检验检疫、海事、地震、气象、水文、消防、邮政、通信、社会科学、决策咨询、法律援助、老龄、残疾人、红十字等各项工作取得新进展，工会、共青团、妇联、工商联等群团组织在经济社会发展中发挥重要作用，国防教育深入开展，国防动员和民兵预备役建设不断加强，双拥共建取得新成绩。

各位代表！“十二五”时期是我市爬坡过坎、克难前行的五年，是强基固本、集聚后发优势的五年，是富民强贺、跨越发展的五年。经过“十二五”的发展，我市正加快向着与全国、全区同步全面建成小康社会的目标迈进。“十二五”的发展证明，必须坚决贯彻落实中央和自治区部署，立足市情，调结构、护生态、壮产业；必须坚持改革开放带动，以更大力度向东开放，抓机遇、增动力、添活力；必须坚持主动适应和引领新常态，创新工作方式方法，增强预见性、主动性、实效性；必须坚持问题导向，集中力量，抓关键、破瓶颈、补短板；必须坚持稳增长惠民生，统筹城乡协调发展，缩差距、优布局、推共享，不断加快爬坡过坎迈上新台阶，砥砺前行实现新目标。

各位代表！五年的成就来之不易。这是自治区党委、政府和市委正确领导的结果，是全市各族人民团结奋斗、顽强拼搏的结果，是历届党委、人大、政府、政协打下的良好基础和各界朋友大力支持帮助的结果。在此，我谨代表市人民政府，向努力工作在各条战线上的广大干部群众，向各位人大代表、政协委员、离退休老同志，向各民主党派、工商联、无党派人士、各人民团体，向驻贺部队、武警官兵，向来贺州创业的投资者和劳动者，向所有关心支持贺州发展的社会各界人士，表示衷心的感谢并致以崇高的敬意！

在取得成绩的同时，我们也清醒地认识到，我市面临的挑战仍然复杂严峻。主要是经济总量小、人均水平低，新常态下实现经济持续稳定增长任务繁重；基础设施欠账较多，行政效能还不够高，基本公共服务发展滞后，补齐民生短板和脱贫攻坚任务艰巨；资源环境约束趋紧，土地、人才、资金、技术等要素供需矛盾突出，区域竞争更加激烈；县域经济发展不均衡，经济开放性水平不高，等等。对此，我们要始终保持清醒头脑，不畏艰难，勇于担当，不断克服困难，认真解决问题，决不辜负全市人民的厚望！

二、“十三五”时期的奋斗目标和主要任务

“十三五”时期，是我市贯彻落实“四个全面”战略布局，与全国、全区同步全面建成小康社会的决胜期，是全面履行自治区赋予我市新定位新使命，建成广西对接东部和中部地区的重要门户和枢纽的关键阶段，是加快爬坡过坎，打造贺州发展“升级版”的重要时段。在前进的道路上，我们要紧紧把握我市发展新定位和国家加快建设“一带一路”“珠江—西江经济带”、推进结构性改革、支持西部地区发展、加快脱贫攻坚，以及贺州“多规合一”试点改革红利全面释放等重大机遇，着力优化经济结构、增强发展动力、化解突出矛盾、补齐发展短板，以更大的开放力度、更优的服务，有效应对各种风险挑战，奋力开创“十三五”发展新局面，全面打响“生态贺州·长寿胜地”的品牌。

市委三届六次全会提出，我市“十三五”时期发展的总体要求是：高举中国特色社会主义伟大旗帜，全面贯彻党的十八大和十八届三中、四中、五中全会精神，以马克思列宁主义、毛泽东思想、邓小平理论、“三个代表”重要思想、科学发展观为指导，深入贯彻习近平总书记系列重要讲话精神，坚持推进“四个全面”战略布局，坚持发展第一要务，全面践行“创新、协调、绿色、开放、共享”五大发展理念，深入实施创新驱动、绿色崛起、向东开放、人才兴贺、新型城镇化“五大战略”，全面推进基础设施建设、支柱产业超千过百、全面脱贫、重点镇建设、美丽贺州乡村建设“五大攻坚行动”，统筹推进经济建设、政治建设、文化建设、社会建设、生态文明建设和党的建设，把贺州建成广西对接东部和中部地区的重要门户和枢纽，奋力与全国全区同步全面建成小康社会。

根据市委的建议，市政府在广泛征求各方面意见的基础上，制定了《贺州市国民经济和社会发展第十三个五年规划纲要（草案）》〔以下简称《纲要（草案）》〕。《纲要（草案）》共分3大板块14篇58章。导语和第一篇构成第一板块，分析“十三五”发展的基础和环境，明确指导思想和发展目标。第二篇至第十三篇构成第二板块，就经济社会发展各领域进行全面部署。第十四篇为第三板块，提出保障措施。在这里着重报告三个方面内容：

（一）关于“十三五”经济社会发展目标。

“十三五”时期我市经济社会发展主要指标，包括经济发展、转型升级、民生改善、生态文明、土地利用5大类33项具体指标，其中最关键的综合性指标就是“超千过百十以上”，即“十三五”期末地区生产总值突破1000亿元，财政收入突破100亿元，GDP年均增长10%以上。城镇居民人均可支配收入、农民人均纯收入年均增长10%以上，城乡居民人均收入比2010年翻一番以上。提出这些目标，主要基于以下考虑：一是全面建成小康社会的需要。根据测算，我市地区生产总值实现翻番，“十三五”需年均增长6.5%，城镇居民人均可支配收入、农民人均纯收入分别需年均增长7.5%和4.5%。二是缩小与全区平均水平差距的需要。当前，我市仍是全区唯一GDP不超过500亿元、财政收入不超过50亿元的地级市，后发展欠发达特征明显。“十三五”期间，自治区把生产总值增速初步定在7.5%以上，如果我市按照年均6.5%的增速发展，虽然可以实现翻番要求，但人均生产总值和居民人均收入与全区平均水平的差距将会进一步拉大。要缩小与全区其他地市的差距，必须保持经济发展增速高于全区一定水平。三是有实现较高增速的良好条件。根据测算，“十三五”期间我市经济潜在增长率为11%左右。从供给方面看，一产年均增长3%，二产年均增长17%，三产年均增长10% ；从需求方面看，固定资产投资年均增长12%，社会消费品零售总额年均增长8%，都是有把握实现的；从居民收入水平来看，“十三五”随着收入倍增计划深入实施、消费升级加快和消费领域拓展、脱贫攻坚任务完成，我市城镇居民人均可支配收入、农民人均纯收入年均增长10%以上也是可以实现的。同时，提出GDP年均增长10%以上目标，有利于增强各方面发展的信心，有利于化解过剩产能、优化产业结构、强化创新驱动，为转型升级提质增效、推进结构性改革留出空间，实现更有质量、更高效益和可持续的发展。

（二）关于深入实施五大战略。

为增创发展新优势，实施创新驱动、绿色崛起、向东开放、人才兴贺和新型城镇化五大战略。

创新驱动战略。加快推进贺州创新基地和一批重点实验室建设，搭建联合科技攻关平台，建成同济·银城产学研基地、高新技术服务中心、科技企业孵化器等重点科技服务平台，以及4个科技企业孵化器、1个大学科技园。实施“大众创业、万众创新”工程，建立创业创新引导基金，鼓励企业及社会机构创办众创空间、大学生创业示范基地、小微企业创业孵化基地，累计孵化创业企业100家以上。推动“项目投资滚动三年计划”项目库建设。发挥政府引导资金及国家各类专项建设债券资金作用，积极稳妥推进政府与社会资本合作（PPP）项目，支持企业通过合法途径积极筹措资金加大技改力度。

绿色崛起战略。以推进国家生态保护与建设示范区建设为契机，实施生态保护与建设工程等生态文明五大建设工程，争创国家生态文明试验区。大力推进生态经济建设，重点抓好生态产业园、循环经济环保产业园和黄姚古镇旅游文化产业区建设，抓好旺高工业区（广西碳酸钙千亿元产业示范基地）等园区生态化改造，支持节能环保产业园、新能源汽车产业化基地建设，规划建设一批种养结合的循环型生态农业示范园。实施生态健康产业27个重点建设项目，做大做强生态健康旅游、生态健康休闲、生态健康养生和生态健康晚年等四大业态。以黄姚古镇、临贺故城等历史文化名城（镇）为核心，发展一批特色旅游名镇名村。加快打造贺州生态水城，推进“一江九河连七湖”中心城区生态水系建设。

向东开放战略。积极推进与广东肇庆共建粤桂产业合作示范区，争创粤桂黔高铁经济带试验区（广西园），创建“一带一路”国际合作工业生态示范园区，着力打造华南旅客集散中心，加快建设贵广高铁经济带先行示范区。全面实施面向粤港澳的招商引资引智工程，引进珠三角中心城市重点产业行业协会等市场主体。加快交通物流体系对接，重点推进永贺高速、洛湛铁路（永州至玉林段）扩能改造、贺州火车站综合交通枢纽等重大交通工程建设，构建桂粤湘区域性综合交通枢纽和物流中心。同时，加快与湖南等地共建潇贺古道旅游文化经济带，打造广西连接西南中南地区经济走廊的重要节点。

人才兴贺战略。围绕碳酸钙、新型建筑材料、生态健康和现代特色农业等产业领域，建设贵广高铁经济带上重要的人才聚集地和面向珠三角为重点的区域性人才小高地，抓好干部交流和双向挂职，引进创新创业人才1200人以上。鼓励企业进行科研创新，激发企业发挥引才引智的主体作用。谋划激励本土人才成长发展的有效举措，完善引导人才向艰苦偏远地区和基层流动的激励机制，有计划地推动一批优秀人才向基层一线重要岗位流动。

新型城镇化战略。继续通过产业和人口集聚，合理扩大城市核心地区的发展规模，提升综合实力，增强辐射带动力。到2020年，市中心城区建成区面积85平方公里，建成区人口达到70万人以上。完成“多规合一”试点工作，引领城镇有序规划建设。加快形成新型市域发展空间，构建以八步区和平桂管理区为核心，钟山、富川、昭平和信都为副中心的“一主四副”空间布局。大力扶持培育17个重点镇。完成“美丽贺州”活动建设任务，加强农村基础设施建设，建立健全运营管理长效机制。

（三）关于“十三五”重点任务。

为突破发展瓶颈，突出抓好基础设施、现代产业、脱贫攻坚、公共服务和改善民生等重点任务。

构建比较完善的现代化基础设施体系。打造 “8421”公路交通圈，建成并开通永贺高速（麦岭至贺州段）、贺州至巴马（钟山至昭平段）等高速公路，规划建设二广高速公路广东连山至广西贺州支线、信都至梧州高速公路等项目，公路总里程达到5400 公里，二级以上公路比重达到39%，通三级以上公路的乡镇比例达到100%，乡镇客运站覆盖率达到100%。打造“4321”铁路交通圈，争取建成洛湛铁路电气化改造项目，开工建设柳州经贺州至韶关铁路和贺州至梧州城际铁路，推进贺州火车站综合交通枢纽和钟山西站综合客运枢纽建设，加快完善富川火车站基础设施，推进信都火车站建设。建设内河水运网络，推进贺江扩能工程和桂江航道工程建设，建成桂江航道工程（平乐至莲花大桥）贺州段93 公里IV 级航道工程。建设贺州支线机场，以及支线机场至城区和各县区的快速交通集疏运通道。完善能源保障体系，重点加快推进华润（富川）火电二期和高纯铝电子动力车间项目建设，积极推动金鸡顶抽水蓄能电站建设，突出做好风电入网工作，加快推进县县通天然气工程和农村生态能源建设，扎实推进“煤改气”“油改气”工作。同时，加强水利设施建设，加快推进“互联网+”与信息服务业融合发展等。

构建竞争力强的现代产业体系。把改造提升传统优势产业与培育发展新兴产业有机结合，推进机械制造业、铝电子产业二次创业，打造成为广西乃至全国重要的铝电子产业基地；实施食品、有色金属与木材加工等传统优势产业改造提升工程; 发展稀土及稀土功能材料、有色金属精深加工、钨基合金及超硬材料等产业；推进“互联网+工业”行动，加快新一代信息技术与传统工业深度融合。发展壮大“两个千亿元产业”，加快推进旺高工业区（广西碳酸钙千亿元产业示范基地）等平台建设和广西旭腾实业集团新型建筑材料产业化示范基地等一批重点项目建设，力争碳酸钙产业和新型建筑材料产业产值分别达600亿元、450亿元以上。提升发展现代服务业，实施12项生产性服务业重点项目，发展壮大特色农产品展销会、中国·贺州国际石材展等展会，科学规划布局全市农贸市场，建设完善市、县、镇、村各级骨干市场。全面统筹发展生态健康旅游、生态健康休闲、生态健康养生、生态健康晚年等四大业态，打造国内知名的生态养生长寿胜地。重点建设黄姚古镇旅游区、姑婆山（路花）旅游区、南乡（西溪）温泉旅游区、大桂山旅游区四大旅游片区，构建“快旅慢游”服务体系，推进桂贺旅游一体化建设，共同打造区域联动的旅游精品路线，建成华南地区高品质的生态健康度假旅游目的地，力争生态旅游业年均增长15%以上。推动特色农业提质增效，推动农业发展标准化、产业化，实施大面积增粮增收工程、有机大米生产基地、“沃土工程”等项目建设，到2020年全市粮食总产稳定在72万吨以上，良种覆盖率保持在95%以上；加快富川神仙湖果蔬产业（核心）示范区等五大现代特色农业（核心）示范区建设，创建国家现代农业示范区；加快“互联网+特色农业+旅游业”发展，打造面向珠三角的现代有机农产品物流中心；加快推进八步蔬菜、平桂马蹄、钟山贡柑、富川脐橙、昭平茶叶等养生长寿食品品牌建设；实施林业发展五大重点工程，确保全市森林保有量89.7万公顷，森林覆盖率达72.92%。

大力推进精准扶贫精准脱贫。到2019年，全市5个贫困县（区、管理区）分4批脱贫摘帽：2016年八步区脱贫摘帽；2017年富川瑶族自治县脱贫摘帽；2018年钟山县脱贫摘帽；2019年昭平县、平桂管理区脱贫摘帽。到2020年，全市267个贫困村分5批全部脱贫出列：2016年脱贫出列20%；2017—2019年每年脱贫出列25%；2020年脱贫出列5%。

持续改善民生福祉。推进教育均衡化发展，继续深入实施学前教育三年行动计划，实现学前教育毛入学率达85%以上；加快推进义务教育学校标准化建设，实现义务教育巩固率达95%以上；加强普通高中基础能力建设，实现高中阶段毛入学率达90%以上；推进贺州学院转型发展，完成学院应用型大学建设工程和大学科技园区、黄姚校区等建设项目；整合贺州职业教育资源，力争达到五年一贯制中高职院校办学标准；加大八步信都工业区农民工创业园建设项目、平桂管理区农民工创业园建设试点项目等重点园区项目实施力度。推进健康贺州建设，实施市人民医院城东分院建设等九大医疗卫生事业发展重点项目，统筹推进医药卫生体制改革，实施基层和全科医生人才培育工程，建立二级以上医院定点帮扶边远山区卫生院机制，积极发展中医药、壮瑶医药等民族医药事业。健全社会保障体系，完善以低保为核心的社会救助制度，全面实施临时救助制度，推进并加强重大疾病医疗救助等专项救助制度建设，建立城乡一体的社会保障体系。完善社会福利制度和残疾人权益保障制度，积极发展社会慈善事业，做好优抚安置工作。统筹推进扶老、助残、救孤、济困等福利事业发展。促进文化事业大发展，重点实施市文化中心、图书馆、博物馆、群众艺术馆、档案馆和贺州学院族群文化博物馆等文化事业项目。大力发展公共体育，扶持发展全民健身公共服务业。积极发展文化创意产业，加大对临贺故城、黄姚古镇、秀水状元村等历史文化古迹、古村落的保护和开发；加快黄姚文化旅游产业区建设，打造自治区级文化创意产业集聚区。2020年，文化及相关产业增加值占GDP比重达5%左右。实现更加充分、质量更高的就业，实施公共就业创业服务设施建设、大学生实训基地、农民工培训实训基地等创业就业工程，城镇调查失业率控制在4.8%以内。深化收入分配改革。实施城乡居民收入倍增计划，增加低收入者收入，扩大中等收入群体，逐步缩小城乡、区域和行业收入差距。

各位代表！我市“十三五”发展的宏伟蓝图已经绘就，跨越发展新的航程已经开启。全市上下团结一心、苦干实干，一定能在全面建成小康社会、建成广西对接东部和中部地区的重要门户和枢纽的道路上阔步前进，开创更加美好的明天！

只要坚持，梦想总是可以实现的。

三、2016年的主要目标与重点工作

今年是实施“十三五”规划的开局之年，做好今年的政府工作意义重大、影响深远。我市发展的主要预期目标是：地区生产总值增长9%以上，财政收入增长10 %，规模以上工业增加值增长8%，固定资产投资增长12 %，社会消费品零售总额增长 8 %，居民消费价格涨幅控制在3%以内，城镇居民人均可支配收入、农民人均纯收入分别增长10%和11%，城镇登记失业率控制在4.2%以内，城镇新增就业1万人，常住人口城镇化率45%，完成自治区下达的节能减排指标任务。确定上述目标，既是从积极稳妥、统筹兼顾、留有余地、与全国全区同步全面小康要求相衔接等角度综合考虑的，也是从贺州市情来考虑的。在实际工作中，我们将全面贯彻落实党的十八届五中全会提出的“五大发展”理念，真抓实干、克难攻坚，力争实现更好的结果，为实现“十三五”发展目标打下坚实基础。为此，重点抓好以下八个方面的工作：

（一）持续加大项目建设力度，不断增强投资拉动力。

强化基础设施建设。重点推进交通、能源、城建和农林水利等基础设施建设，为经济发展提供有力保障。交通方面：全面打赢2014—2016年综合交通基础设施建设大会战。扎实谋划铁路建设，重点力争完成柳州经贺州至韶关铁路、贺州至梧州城际铁路、洛湛铁路（永州至玉林段）扩能改造等项目前期工作，抓好贺州火车站、信都火车站等站房改扩建。全力推进公路建设，力争年底永贺高速公路贺州段全线贯通，实现黄姚景观大道、昭平富裕至马江等项目建成完工，推进贺州至巴马高速公路（钟山至昭平段）、贺富一级公路北环路段（莲塘至西湾段）等项目建设。积极建设内河水运，推进桂江航道工程前期工作，实现贺江（八步至厦岛段）航道整治工程竣工。稳步推进贺州机场项目前期工作。能源保障方面：加快建设八步天堂明梅等风电场和桂东电力立头、扶隆等220千伏输变电工程，开工建设垃圾焚烧发电项目，推进电源建设、电网改造、县县通天然气等项目相关工作。农林水利方面：稳步推进中小河流治理工程建设工作，加快实施贺江、桂江主要支流整治工程。做好良佑、横塘等病险水库除险加固工程建设和竣工验收。实施农村饮水安全提质增效工程，完善县级水质检测中心机构建设。推进小农水重点县建设和大中型灌区续建配套与节水改造。

解决项目推进难题。集中力量啃下征地拆迁“硬骨头”，打通审批程序“快车道”，全面提升项目建设水平。全力推进项目征地拆迁工作，重点解决2003年以来中心城区内征地拆迁遗留问题，对“批而未供”和闲置土地开展节约集约用地专项清查整改，确保我市近五年已批建设用地土地供应率在60%以上。重点确保中央投资、工业园区、农村建房和保障性安居工程、学校、交通基础设施等五类项目的用地需求。把落实农民“两地”作为征地拆迁工作的前置任务，研究出台货币补偿、土地回购和失地农民社保等政策。切实提高项目审批效率，进一步完善项目集中联合审批制度，建立并联审批工作机制，提高规划审批、土地预审、环境评估等程序效率。

拓宽项目投融资渠道。制定实施贺州市2016—2018三年项目滚动投资计划，完善重大项目储备库。稳步推广运用政府和社会资本合作（PPP）模式，加快推进黄安寺、狮子岗排洪截污工程等PPP备选项目建设。用好用足政府债券、农发行、国开行等金融部门贷款及国企发债融资，重点支持保障性住房、城市基础设施等项目建设。稳步实施工程总承包试点，积极探索公益性、基础性政府投资项目建设模式。推进政府购买服务，支持非公有制经济加快发展，激发和扩大民间资本投向项目建设。同时，做好投资存量资金清理和调整使用工作。

（二）积极推动转型升级，全面强化工业发展引擎。

继续强化科技创新。大力推动“大众创业、万众创新”，深化与中国科学院等科研院所合作，推进碳酸钙研究开发与应用、微波应用技术等重点实验室建设，共建创新驱动基地。加快粤桂县域经济产业合作示范区综合型孵化器等建设步伐。推进贺州国家级农业科技园区和自治区级贺州高新技术产业开发区建设，打造生态科技服务集聚区。组建碳酸钙产业人才小高地，建设桂东电子博士后科研工作站。支持企业技术中心和工程技术研究中心建设，提升电子信息、新型建筑材料、碳酸钙和食品药品产业的核心竞争力。

力促传统产业升级改造。我市发展旧的动力如钢铁、机械、铝电子等产业板块体量较大，当前乃至今后一段时期对经济增长仍具有重要支撑作用。要抓紧实施新一轮技改工程，重点支持兆鑫钢铁、长城矿山机械等一批骨干项目技术改造。鼓励开展铝电子等产业“二次创业”，大力推进桂东铝电子产业项目，发展精铝、电子铝箔、铝电解电容器等产业，打造特色铝产业链循环经济发展模式。

发展壮大“两个千亿元产业”。积极培育新产业、新业态，扎实推进碳酸钙基地人造岗石产业园、新型建筑材料产业园等“园中园”建设，重点推进贺州续宝、广西旭腾纳米科技板、超超新材料、旺高新型材料交易中心等项目建设，力争碳酸钙产业年总产值突破150亿元。同时，以贺州生态产业园、华润循环经济产业园为平台，支持发展新能源、新材料和健康食品药品等产业，加快华润循环经济产业园区二期、景鸿新能源汽车产业园等项目建设。

加快完善工业园区基础设施。围绕提升园区的承载力和集聚力，开展“园区建设推进年”活动，完善园区水、电、路、排污及标准厂房等基础设施，积极创建国家级产城融合示范区。重点推进碳酸钙展示长廊、粤桂县域经济产业合作示范区工业大道、生态产业园标准厂房等建设，确保园区基础设施投资增长30%以上。

（三）坚持“生态立市”，全力打造 “生态贺州•长寿胜地”。

推进生态城镇规划建设。加快 “多规合一”试点工作，统筹推进智慧城市建设。抓好“生态新城” “贺州生态水城”和“一江两岸”建设，加快推进北堤公园提升改造、大钟山公园等项目，启动建设第十届广西（贺州）园林园艺博览会参展城市和国际友好城市展园。深入实施市容市貌综合提质三年行动，推进贺州市旧城区管网改造、贺州市污水处理厂扩建和各乡镇污水处理厂建设，实现黄安寺、狮子岗、排洪河全面截污目标，加快乡镇自来水厂建设和集中供水管网升级并网改造。继续实施“大县城”战略，支持打造各具特色的美丽县城。推进17个重点镇规划建设，加快城乡一体化进程。

加强生态保护与建设示范区建设。持续开展大气污染专项集中整治行动，推进城乡集中式饮水水源地保护工程。对龟石国家湿地公园、爱莲湖生态湿地公园等实施退养还湿、生态修复。抓好龙水金矿和西湾、黄田等老矿区环境治理。实施新一轮退耕还林、生态公益林保护、石漠化综合治理等林业生态工程，年内造林面积20万亩。全面推进“生态乡村”建设，深入实施“村屯绿化”“饮水净化”“道路硬化”“村屯整洁化”专项活动和“五改”工程，打造自治区级绿化村屯323个，一般绿化村屯2575个。

高效优质发展生态健康旅游业。以黄姚古镇旅游区、姑婆山（路花）旅游区、南乡（西溪）温泉旅游区、大桂山旅游区等四大板块为抓手，打造包括生态健康旅游、生态健康休闲、生态健康养生、生态健康晚年的大旅游产业，塑造“山清水秀人长寿”的贺州形象。推进昭平特色旅游名县建设，抓好姑婆山国家森林公园、黄姚古镇景区创建国家5A级景区工作，争创“国家旅游标准化示范城市”。大力发展一批星级休闲农家乐项目。加快建设西溪森林温泉度假村、仙女湖休闲度假区、黄姚水街、梦里黄姚文化旅游创意城等项目，争取落地实施贺州古城、大商汇等一批重点项目，确保游客接待量增长14.8%，旅游总收入增长21.6 %。加快发展生态健康长寿产业，研究出台《贺州市建设休闲旅游养生晚年产业示范区实施方案》，力争贺州社会医养综合服务示范项目、华南民族医疗康复中心等项目列入自治区养老服务业综合改革试验区规划并实施，打造国内知名的生态养生长寿胜地。

加快发展生态特色农业。积极创建自治区级现代特色农业核心示范区，推进国家农业科技园区建设。重点支持发展富硒农业、生态有机循环农业和生态健康水产业，谋划建设一批度假观光、农业休闲体验项目。加快完善农产品配送中心和交易市场，构建“1+5”农产品物流市场体系。强化农业公众品牌培育，支持农产品网上交易，加快发展农村电商。强化粮食安全责任，大力实施粮食稳定增产增效行动，确保粮食种植面积稳定在206万亩以上，粮食产量稳定在72万吨以上。稳定农村基本经营制度，基本完成全市土地确权登记颁证工作。

（四）努力推进结构性改革，进一步释放发展活力。

推动供给侧改革。面对经济下行压力，必须优化存量、引导增量、有效减量，加快去产能去库存，全面提高效率。努力增加有效供给，通过大力发展实体经济、增加有效投资、补齐软硬基础设施短板，推动产业向中高端迈进。同时，大力实施扶优扶强计划，推动主营业务收入1000万元以上、2000万元以下的企业尽快进入规模以上企业行列。降低企业成本，落实中央、自治区的结构性减税方案，清理和规范涉企行政事业性收费，加强中介机构改革和收费监管。特别是，着力降低大工业电价，力争华润电力向桂鑫钢铁等用电大户直供电纳入自治区第二批直供电交易试点。抓好去产能去库存，继续加大对钢铁、水泥、冶炼等行业的落后产能淘汰力度。积极推进保障性安居工程货币化安置与“以购代建”模式相结合，加快棚户区和危旧房改造，加快消化商品房库存。

深入推进简政放权。完善和运用“多规合一”业务协同平台，提升项目审批效率。加强市县政务服务网上平台建设，实现串联改并联审批。做好行政审批事项“接放管”工作，年内，涉及区域管理外的其他事项最大限度下放县区办理。市县两级7月底前全面依法公布权力清单和责任清单。深化商事制度改革，全面落实“先照后证”，巩固和扩大“三证合一、一照一码”改革成果。大力推进审批服务“两个即办”工作。

加快行政体制改革。进一步完善国企营运机制，稳步开展党政机关、事业单位与所属企业脱钩工作。统筹推进事业单位分类改革。推进行政综合执法体制改革试点工作，在文化旅游、交通运输等领域开展综合行政执法体制改革试点。进一步理顺工业区（产业园区）体制机制，激发园区发展活力。加快平桂管理区设区步伐，积极谋划成立信都新区。

（五）加快“向东开放”步伐，全面融入珠三角经济圈。

强化平台建设与产业合作。以建设贵广高铁经济带先行示范区为引领，加快粤桂县域经济产业合作示范区合作共建，创建“一带一路”国际合作生态工业示范园区。紧密对接广东自贸区，争创粤桂黔高铁经济带合作试验区（广西园）。大力推动纯一生态陶瓷、骏华五彩装饰板等项目竣工运营，确保实现示范区工业总产值突破200亿元。积极发展“互联网+旅游”新模式，加强与桂林、港澳以及佛山、肇庆等地旅游交流合作，持续打响“坐上高铁去贺州”品牌，融入桂粤黔大旅游区建设。强化供港澳蔬菜基地建设，出口蔬菜15万吨以上。围绕生态健康和“两个千亿元产业”等重点产业，谋划一批优惠政策和精品项目，吸引发达地区更多知名企业合作入驻。

加快完善对外开放格局。积极参与“一带一路”“珠江—西江经济带”等建设，重点推进交通、城市和园区等基础设施项目建设，加快打造广西对接东部和中部地区的重要门户和枢纽。围绕重点园区和重大产业布局，加快建设跨省交通项目，积极发展“铁海江”联运，着力打造现代物流集聚区。大力实施果蔬、茶叶、碳酸钙产品等一批物流项目，加快富川信立特色农产品加工物流中心等项目建设，开工建设贺州石材碳酸钙会展中心，支持旺高新型材料交易中心创建自治区级现代物流集聚区。

（六）开展精准脱贫攻坚，如期实现脱贫致富目标。

全面完成精准识别建档立卡工作，加快建立扶贫大数据管理平台。完成“十三五”扶贫开发规划和市县两级扶贫移民搬迁、扶贫产业开发等专项规划编制工作。加大预算资金、存量资金投入，用足用好自治区今年整合资金及重点用于贫困县的专项资金。扎实推进脱贫攻坚“1315”工程，力争减少贫困人口7.6万人，实现53个贫困村脱贫出列，八步区脱贫摘帽。落实并开工建设八步南乡、钟山扶贫生态移民产城融合示范工程等项目，大力推进平桂、黄姚镇黄姚街、古城镇大岭村等57个扶贫生态移民项目建设，搬迁人口2.62万人。

（七）持续保障和改善民生，切实提升人民生活质量。

加快推动教育振兴。加快实施“全面改薄”工程项目，全面改善158所义务教育薄弱学校基本办学条件。加快推进贺州第二高中扩建、市体育运动学校等教育项目建设。扎实推进第二期学前教育三年行动计划，推进义务教育学区制管理改革，做好消除义务教育学校“大班额”“大通铺”攻坚工作。支持贺州学院黄姚校区建设，加快推进贺州职业学院组建后续工作。

扎实推进就业创业和社会保障工作。加快农民工创业园和创业孵化基地建设。实施“大学生就业促进计划”“大学生创业引领计划”。加快社会保险逐步从“制度覆盖”向“人群覆盖”转变。加强社会保险扩面征缴工作，推进“五险合一”社会保险数据大集中系统建设，做好社会保障卡 “一卡通”工作。推进社保经办标准化，建设全区行政服务综合标准化试点。开展“阳光社保”工作，保障社会保险基金安全运行。

加快发展文体卫计等事业。抓好农贸市场升级改造等“十大为民办实事”项目。建成150个服务功能完善的农村新型生态社区。深入实施“千村万户文艺惠民工程”，提升城乡公共文化服务能力。抓好传统文化的挖掘、保护和传承。加快市文化中心、文化“三馆”和村级公共服务中心建设，做好广西第十三届运动会筹备和广西第八届农运会承办工作。加强文化市场监管，深入开展“扫黄打非”各项工作。加快推进富川、钟山县级公立医院改革；推进城乡居民大病保险实现全覆盖，建立疾病应急救助制度。加快推进市人民医院城东分院和市中医医院综合大楼项目建设，完成市妇幼保健院整体搬迁项目前期工作。稳妥实施全面两孩政策，抓好优生健康惠民工程。

营造和谐稳定的社会环境。继续加强社会治理创新，深入推进公安“八区”建设，持续构建社会整体防控体系。加强信访工作，推进信访法制化建设。推动建立市县乡村四级应急广播指挥平台。加强安全生产和食品药品安全综合治理，深入开展安全隐患排查治理和专项整治工作。同时，深入做好救灾救济、优抚安置、社会福利和慈善事业等工作。完善灾害事故预防预警和应急处置体系，全力维护社会安全稳定。

（八）稳步推进财政金融工作，有效支撑经济社会发展。

提升财政工作水平。强化财税收入征管，集中力量扶持支柱产业做大做强，培植壮大财源。严控“三公”经费支出，保持“三公”经费零增长。全面清理结余结转资金，按规定可以收回的一律收回总预算统筹安排使用，重点用于民生项目建设。强化财政监督，建立完善财政资金监管体系。加大对重点领域、重大财政政策等方面资金的检查，确保资金安全。

推动金融创新工作。采取奖励、风险补偿等有力措施，激发金融机构发展的积极性，做大金融业务总量，支持我市现代产业、基础设施、民生保障等重大项目和重点领域建设。用好用足中央、自治区给予的小企业贷款风险补偿和中小微企业信用担保机构风险补偿资金、小额担保贷款财政贴息资金，帮助中小微企业、困难弱势群体拓宽融资渠道。

各位代表！做好今年和“十三五”经济社会发展各项工作，必须进一步加强政府自身建设，尽快提高新常态下推动发展的能力和水平。深入推进依法行政，全面履行政府职能，规范行政权力，强化严格执法，加快建设法治政府。自觉接受人大及其常委会的监督，定期向人大及其常委会报告工作，积极支持人民政协履行政治协商、民主监督、参政议政职能，切实做好人大代表建议和政协提案办理工作。认真听取各民主党派、工商联、无党派人士和各人民团体的意见建议。巩固 “三严三实”专题教育成果，持之以恒纠正“四风”；严格落实党风廉政建设主体责任，履行“一岗双责”，坚持不懈推进政府系统党风廉政建设和反腐败工作。

各位代表！“十三五”和今年经济社会发展任务十分繁重，责任十分重大。让我们团结奋进，开拓创新，真抓实干，为实现全面建成小康社会的宏伟目标而努力奋斗！
